# Parallel and Convergent Evolution of the Dim-Light Vision Gene *RH1* in Bats (Order: Chiroptera)

**DOI:** 10.1371/journal.pone.0008838

**Published:** 2010-01-21

**Authors:** Yong-Yi Shen, Jie Liu, David M. Irwin, Ya-Ping Zhang

**Affiliations:** 1 State Key Laboratory of Genetic Resources and Evolution, Kunming Institute of Zoology, The Chinese Academy of Sciences, Kunming, China; 2 Laboratory for Conservation and Utilization of Bio-resources, Yunnan University, Kunming, China; 3 Graduate School of the Chinese Academy of Sciences, Beijing, China; 4 Department of Laboratory Medicine and Pathobiology, University of Toronto, Ontario, Canada; 5 Banting and Best Diabetes Centre, University of Toronto, Ontario, Canada; University of Stellenbosch, South Africa

## Abstract

Rhodopsin, encoded by the gene *Rhodopsin* (*RH1*), is extremely sensitive to light, and is responsible for dim-light vision. Bats are nocturnal mammals that inhabit poor light environments. Megabats (Old-World fruit bats) generally have well-developed eyes, while microbats (insectivorous bats) have developed echolocation and in general their eyes were degraded, however, dramatic differences in the eyes, and their reliance on vision, exist in this group. In this study, we examined the rod opsin gene (*RH1*), and compared its evolution to that of two cone opsin genes (*SWS1* and *M/LWS*). While phylogenetic reconstruction with the cone opsin genes *SWS1* and *M/LWS* generated a species tree in accord with expectations, the *RH1* gene tree united Pteropodidae (Old-World fruit bats) and Yangochiroptera, with very high bootstrap values, suggesting the possibility of convergent evolution. The hypothesis of convergent evolution was further supported when nonsynonymous sites or amino acid sequences were used to construct phylogenies. Reconstructed *RH1* sequences at internal nodes of the bat species phylogeny showed that: (1) Old-World fruit bats share an amino acid change (S270G) with the tomb bat; (2) *Miniopterus* share two amino acid changes (V104I, M183L) with Rhinolophoidea; (3) the amino acid replacement I123V occurred independently on four branches, and the replacements L99M, L266V and I286V occurred each on two branches. The multiple parallel amino acid replacements that occurred in the evolution of bat *RH1* suggest the possibility of multiple convergences of their ecological specialization (i.e., various photic environments) during adaptation for the nocturnal lifestyle, and suggest that further attention is needed on the study of the ecology and behavior of bats.

## Introduction

Vision plays an extraordinary role in animals and is often basic for their survival. Due to the high degree of variation in light conditions and presence of various wavelengths of light in different environments, the evolution of vision to various photic environments and lifestyles is among the most significant mammalian adaptations [1]. Most mammals have two vision systems, one based on cone photoreceptors and one on rod photoreceptors. The rods are 100 times more sensitive to light than the cones, and are responsible for night vision (dim-light vision) [2], however, they are not sensitive to color. In contrast, cones provide color sensitivity which is due to the presence of two types of cone photoreceptors in most mammals: cones with long/middle wavelength (L/M or red/green) opsin and cones with short wavelength (S or blue) opsin. The size and shape of eyes, photoreceptor organization, and color sensitivity differ dramatically depending upon an animals' specific needs and photic environments [3]. Some primates have undergone one or more duplications of M/L opsin genes, thus have become trichromatic [4], [5], [6]. In contrast, many nocturnal primates, carnivores, and rodents have lost the functional short wavelength opsin, and depend upon rods to maximize their visual sensitivities to the available dim-light rather than to color discrimination [7], [8], [9], [10].

Bats are one of the largest groups of mammals, and all have a nocturnal lifestyle, however, vision is variable among species. Megabats (Old-World fruit bats) rely on vision and olfaction much more than microbats (insectivorous bats), and their eyes tend to be larger and more prominent [11], [12], [13], [14]. Microbats (insectivorous bats) mostly use acoustic orientation (echolocation) rather than vision, and their eyes are generally degraded [15], [16], [17], however, dramatic differences in the sizes and light sensitivity of the eyes of different species of insectivorous bats exist [18].

Rhodopsin, encoded by the gene *Rhodopsin* (*RH1*), known as visual purple, is a pigment in the retina that is responsible for both the formation of photoreceptor cells and the perception of light. Rhodopsins are extremely sensitive to light, enabling vision in low-light conditions. Bats are adapted to a nocturnal niche. Insectivorous bats generally rely upon echolocation to fly and have degraded eyes, while Old-World fruit bats generally navigate by sight, and so have larger eyes and no laryngeal echolocation [19]. This indicates that the dependence upon the visual system varies between species. To examine adaption to a dim-light environment and differences on the reliance on sight, we amplified and sequenced the dim-light vision gene (*RH1*), and two color vision genes (*SWS1* and *M/LWS*) from the total RNA from retina samples from 23 species of bats, to provide a more complete perspective on the evolution of vision in bats.

## Results and Discussion

**Figure 1:**
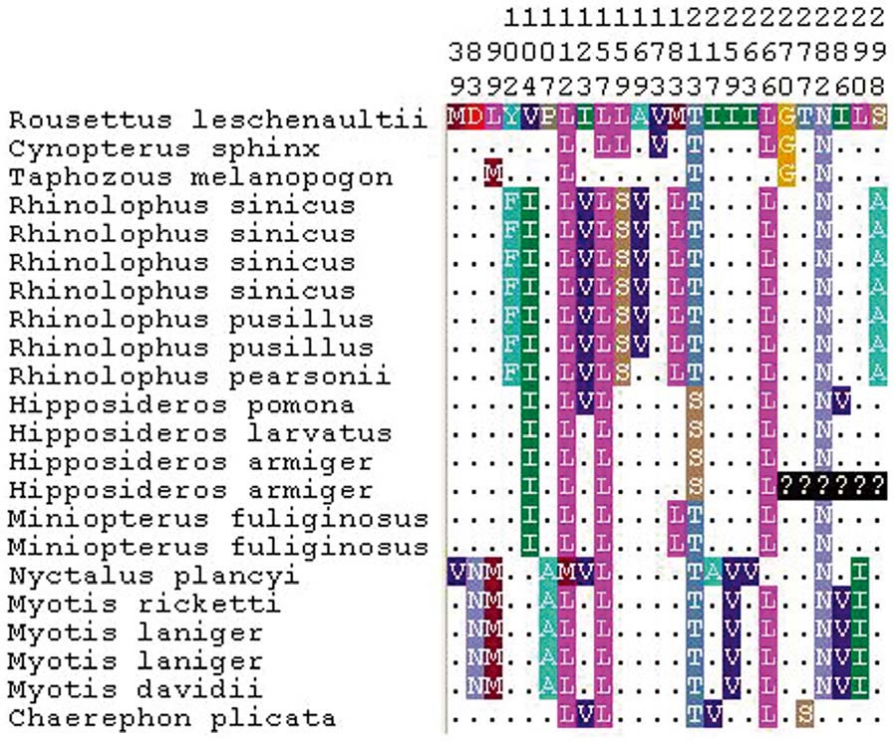
Divergent amino acid sites in the *RH1* gene sequences of bats.

We successfully amplified cDNAs for the rod pigment gene (*RH1*) from retina total RNA for all bats used in this study. The aligned bat *RH1* nucleotide sequence was 834bp in length, of which 147 were variable, but only 24 of these sites cause amino acid sequence variation (Figure 1). No insertion/deletion mutations or changes that result in stop codons were found in the sequences suggesting that all bats have a *RH1* that function in dim-light vision. For the two color vision genes, the *M/LWS* gene was successfully amplified for all bat species (Figure S1), while the *SWS1* gene failed to amplify in all Rhinolophoidea and *Rousettus* species, implying the loss of function of short wavelength opsin in this species, in agreement with a recent study [20].

**Figure 2:**
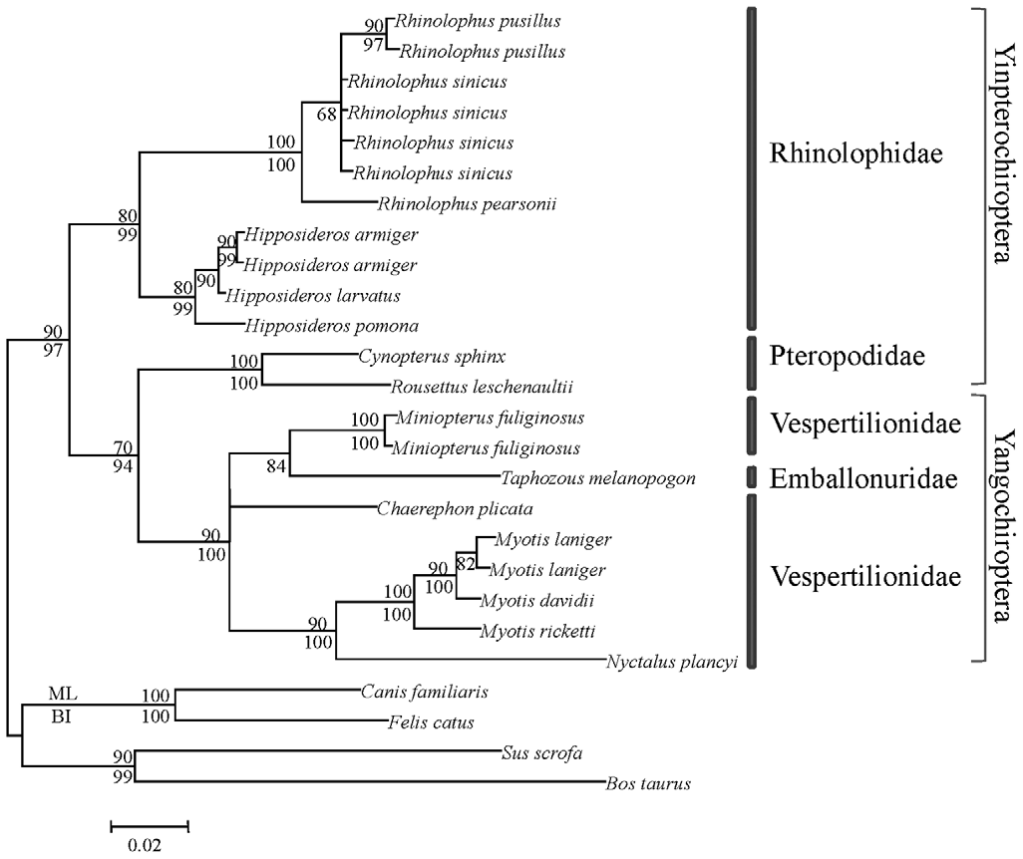
The phylogenetic topology of the *RH1* gene based on nucleotide sequences. Numbers above the branches are the ML bootstrap values, while those under the branches are the Bayesian posterior probabilities.

Phylogenetic analyses of the nucleotide sequences of aligned *RH1* gene with Bayesian and Maximum Likelihood methods revealed that the Pteropodidae (Old-World fruit bats) did not cluster with the Rhinolophoidea, but rather clustered with the Yangochiroptera (Bootstrap values: ML 70; Bayesian 94) (Figure 2). The *RH1* topology differs considerably from the extensively supported consensus tree for these species [21], [22]. Unlike the *RH1* gene, the *M/LWS* opsin gene generates a phylogeny in agreement with the consensus tree (Figure S2). A similar result was obtained when a *M/LWS* phylogeny was generated from the same set of species that was used for the *RH1* tree, indicating that taxon sampling was not the cause for the difference in phylogeny. The topology based on the *SWS1* gene failed to resolve the relationships between Pteropodidae (Old-World fruit bats), Rhinolophoidea and Yangochiroptera (Figure S3), however in contrast to *RH1*, this phylogeny in not in conflict with the consensus species phylogeny (i.e., it did not provide positive evidence for an incorrect species relationship). These results yield the intriguing question: why did the gene tree for *RH1* conflict the species tree?

**Figure 3:**
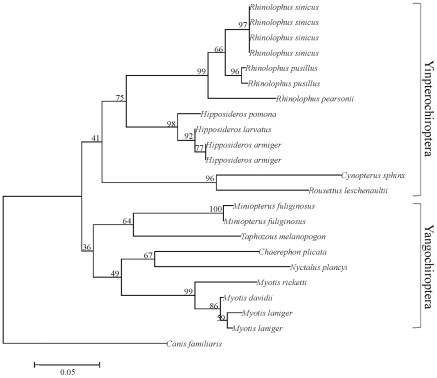
NJ tree based on synonymous sites of *RH1* gene. Numbers above the branches are the NJ bootstrap values.

**Figure 4:**
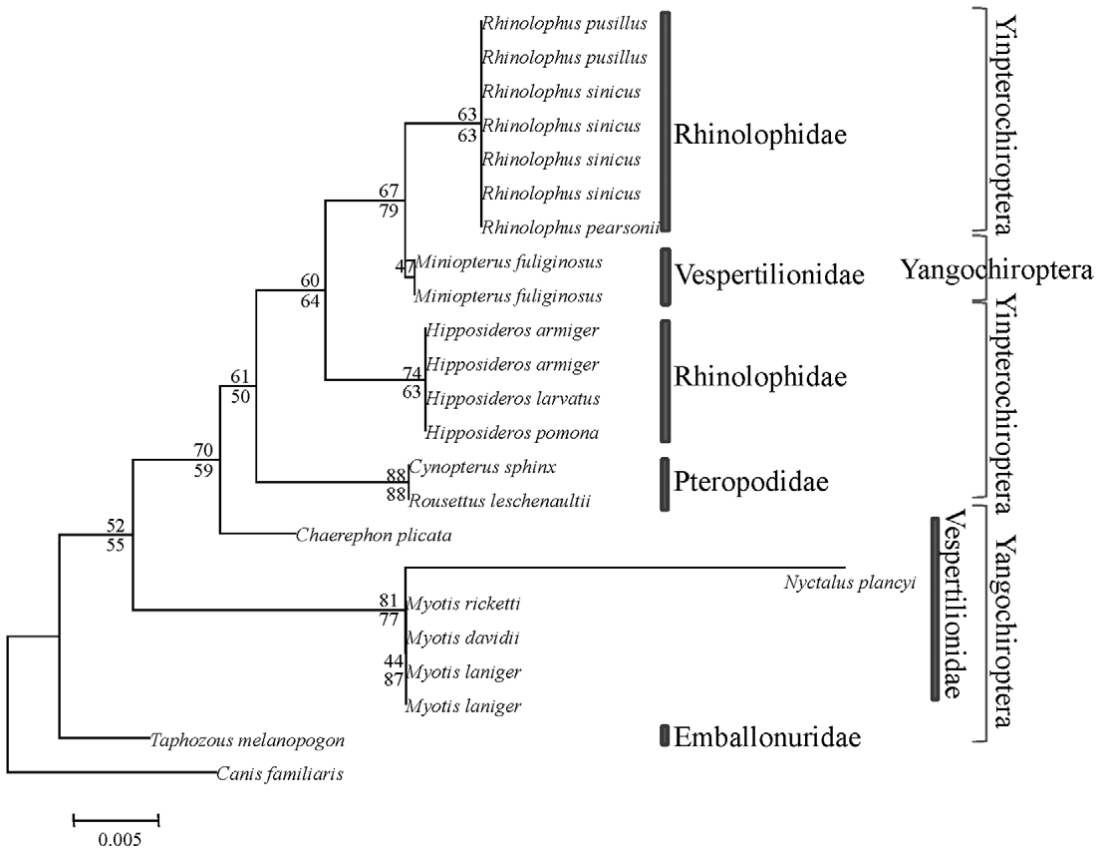
NJ tree based on amino acid sequences and nonsynonymous sites of *RH1* gene. The numbers above the branches are NJ bootstrap values of amino acid sequences, while the numbers under the branches are the values from the nonsynonymous sites.

To further examine this question, we reconstructed the topology of bats using only the synonymous changes in *RH1*, sites which are believed to be without selection. The resulting topology roughly coincided with the expected traditional tree (Figure 3), that is, Pteropodidae (Old-World fruit bats) clustered with Rhinolophoidea forming the Yinpterochiroptera, which was the sister group of Yangochiroptera. This result showed that the synonymous sites in *RH1* were evolving as expected and thus we can exclude the possibility of gene duplication and sequence error caused an erroneous phylogeny and implying that nonsynonymous sites may be confusing the gene tree. This suspicion was confirmed by reconstructing phylogenies using either nonsynonymous changes or amino acid sequences (Figure 4), in both cases, Pteropodidae (Old-World fruit bats) did not cluster with Rhinolophoidea, but rather showed a closer relationship with Yangochiroptera, and *Miniopterus* was not within Yangochiroptera but instead within Rhinolophoidea. Although the bootstrap support values were relative low, likely due to the small number of nonsynonymous or amino acid substitutions that could be used to reconstruct the topology, these low values are expected. An unexpected observation was that the branch length for *Nyctalus plancyi* was very long, but the use of sequences from two additional samples of *Nyctalus plancyi* yielded the same result.

**Figure 5:**
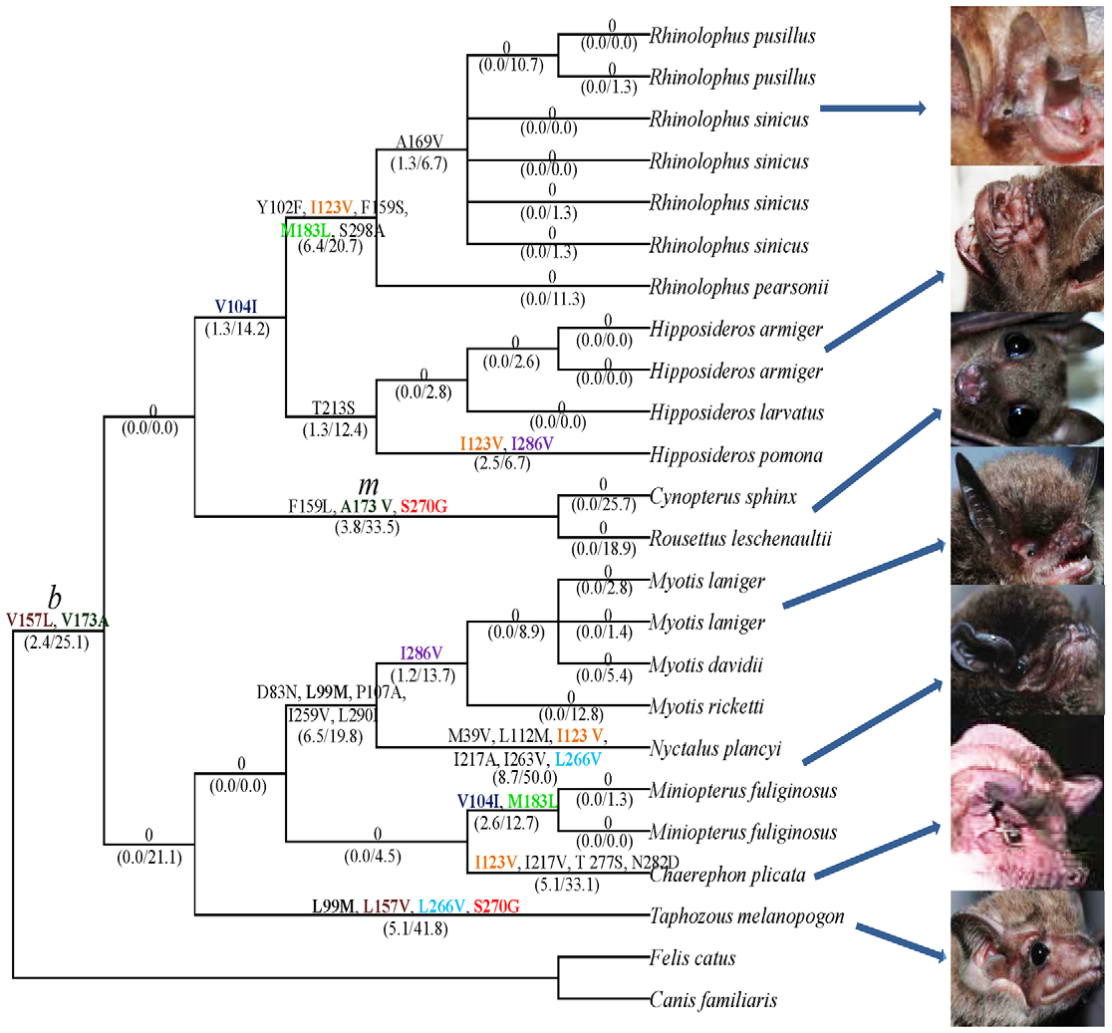
Species tree based on the previous study of Teeling et al. 2005. The numbers and symbols above the branches are the positions and amino acid replacements. The numbers in brackets below the branches are the numbers of nonsynonymous and synonymous substitutions. The sequences of the internal nodes and *Ka/Ks* were reconstructed by Maximum Likelihood method in PAML.

Analyzing ancestral sequences is a powerful method to elucidate the evolution of opsin sequences [23], thus we reconstructed ancestral *RH1* sequences for internal nodes of the species tree and inferred the changes that occurred on each lineage. The lineages leading to Old-World fruit bats and tomb bat both share the S270G (Figure 5, marked in red) amino acid change. Intriguingly, two amino acid substitutions that occurred in the ancestor of all bats, V157L and V173A (Figure 5, marked in coffee and dark green, respectively), have been reversed in the tomb bat and Old-World fruit bats respectively. This pattern of changes is also evident in the aligned amino acid sequences (Figure 1). Parallel substitutions are also observed in other portions of the tree (Fig. 5). Sequences from the genus *Miniopterus* share two amino acid changes (V104I and M183L, marked in dark blue and green in Figure 5) with Rhinolophoidea. The substitution I123V (marked in orange) occurred on four branches, while L99M (marked in black), L266V (marked in light blue) and I286V (marked in purple), each occurred in parallel on two branches (Figure 5).

**Figure 6:**
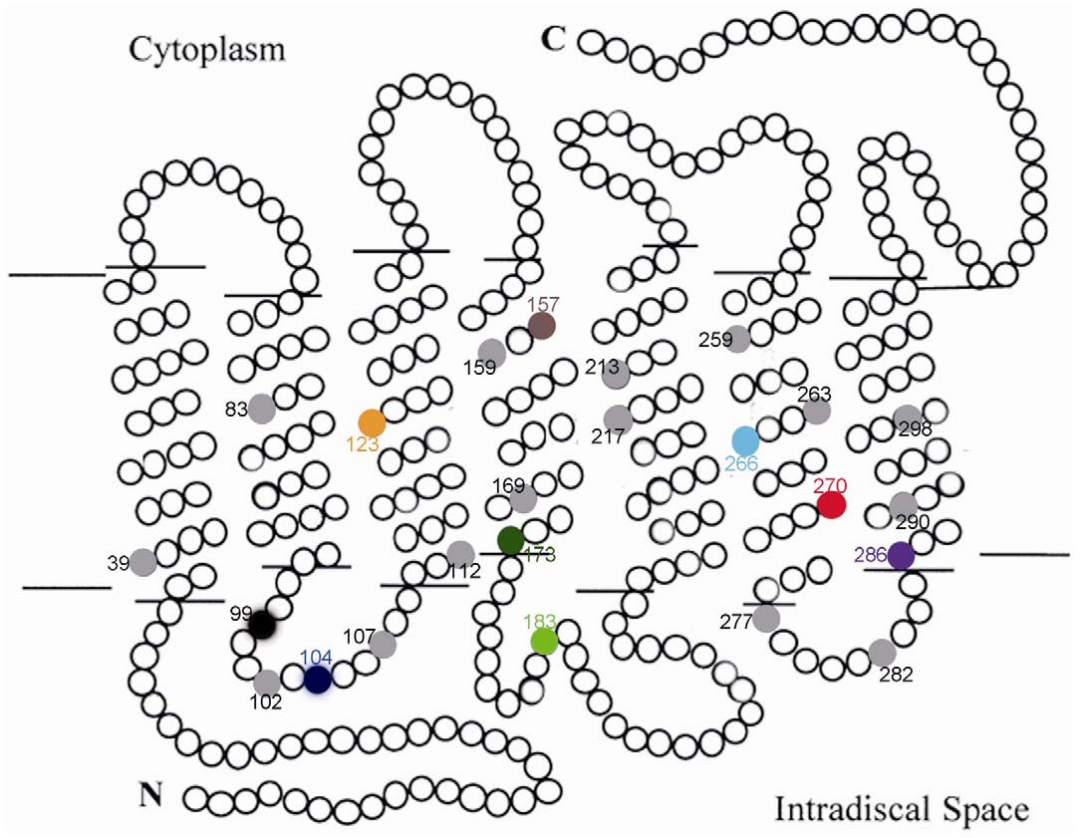
The secondary structure of the *rhodopsin* based on the bovine model [24] with the 24 amino acid replacements among bats identified. Each circle represents one amino acid residue. The numbers around the circles are the positions of the amino acid replacements.

To examine the distribution and consequences of the amino acid substitutions, we mapped all of the amino acid changes in the bat sequences to a secondary structure model based on the structure of the bovine sequence [24]. Of the 24 amino acid changes, 17 mapped to the transmembrane domains, seven of which are in the intradiscal space (Figure 6). None of the substitutions map to the cytoplasm space (Figure 6). Intriguingly, the three of the amino acid changes (S270G, V157L, and V173A) that occurred on the tomb bat and Old-World fruit bat lineages, the lineages that were united in the *RH1* gene tree phylogeny, are all in the transmembrane domains (Figure 6). The location of these three residues suggests that they may have a functional role in vision, and that the amino acid changes may cause shifts the λmax values.

**Figure 7:**
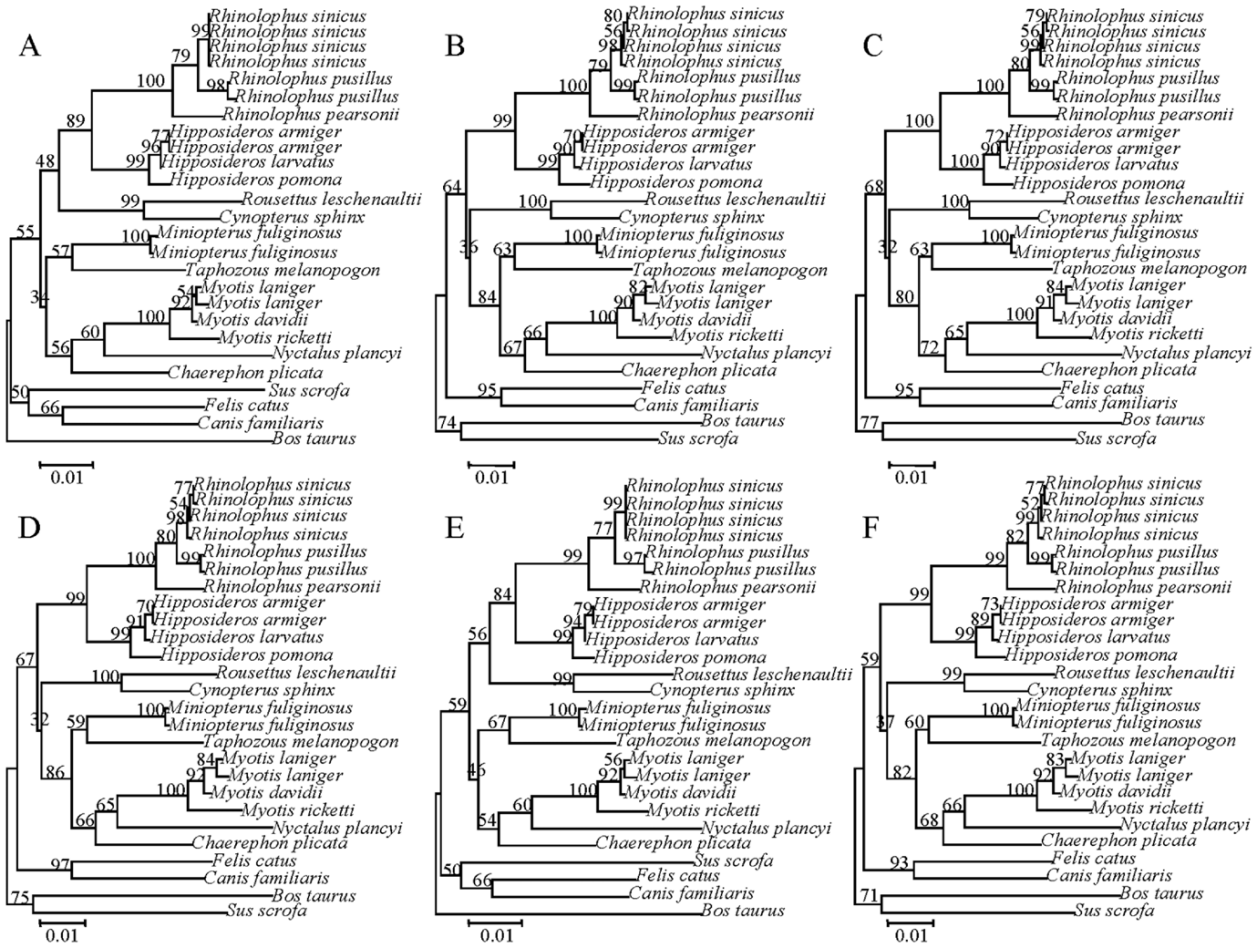
NJ trees based on nucleotide sequences of aligned *RH1* gene, but excluding sites that evolve in parallel. (A) excluding the sites corresponding to amino acid site 270; (B) excluding the sites corresponding to amino acid site 104; (C) excluding the sites corresponding to amino acid site 183; (D) excluding the sites corresponding to amino acid sites 104 and 183; (E) excluding the sites corresponding to amino acid sites 104, 183, and 270; (F) excluding the sites corresponding to amino acid sites 157 and 173.

To determine whether the parallel amino acid substitution are responsible for the *RH1* gene topology, we reconstructed a NJ tree with the nucleotide sequences of aligned *RH1* gene which excluded the sites that showed parallel changes (Figure 7). When we excluded only the nucleotide sites that correspond to amino acid site 270, we attained a tree in rough agreement with the species tree (Figure 7A). Exclusion of the other sites that showed parallel amino acid substitutions did not result in the expected topology, although they did lead to greater support for some parts of the tree (Figure 7B–F). These results indicate that the parallel change at amino acid site 270 was responsible for incorrect species phylogeny in the gene tree, and probably reflects a functional importance for this site.

To examine the selective forces acting upon the *RH1* sequences, maximum likelihood estimates were made for the ratio of nonsynonymous to synonymous substitution rates (*Ka/Ks*) on each branch of the species tree with the PAML package (Figure 5). A low level of variability in substitution rates (*Ka/Ks*) was observed among bat lineages (0.0001–0.2032, PAML M1 model, Table 1). If the M0 model was used, it was observed that the average *Ka/Ks* ratio was 0.0291, which is lower than the value for the *M/LWS* gene is (0.0603 for the same taxon sampling as for *RH1*, 0.0731 for complete samples, Table S1), indicating that both *RH1* and *M/LWS* are experiencing very strong purifying selection in bats, with *RH1* possibly experiencing stronger purifying selection, and that the mutations that have occurred independently on the different branches of the bat phylogeny may have functional importance and not random mutations in inactive genes.

**Table 1:** Selective pressure analyses on *RH1* gene.

Model	P	Ln L	Estimates of parameters				
M0: one ratio	61	−5362.243336	ω = 0.0291				
Two ratio:							
The common ancestor of bats	62	−5362.046108	ω_b_ = 0.0163, ω_0_ = 0.0294				
The common ancestor of bats ω_b_ = 1	61	−5372.736900	ω_0_ = 0.0289				
Megabats	62	−5362.159966	ω_m_ = 0.0225, ω_0_ = 0.0293				
Megabats ω_m_ = 1	61	−5380.586595	ω_0_ = 0.0286				
Site models (only contain bats)							
M1a	41	−2376.845476	p:	0.98192	0.01808		
			ω:	0.01635	1.00000		
M2a	43	−2376.845476	p:	0.98192	0.01130	0.00678	
			ω:	0.01635	1.00000	1.00000	
M8	43	−2372.427477		Pr(ω>1)	post mean +− SE for ω		
			123 I	0.652	1.397+−0.850		
			217 I	0.662	1.396+−0.839		
M8a	42	−2372.416062	p_0_ = 0.98890	p = 0.10800	q = 4.35638		
			(p_1_ = 0.01110)	ω = 1.00000			
Branch-site models							
The common ancestor of bats	64	−5322.454355	site class	0	1	2a	2b
			proportion	0.96419	0.03581	0.00000	0.00000
			background ω	0.02195	1.00000	0.02195	1.00000
			foreground ω	0.02195	1.00000	1.00000	1.00000
The common ancestor of bats ω_b_ = 1	63	−5322.454355	site class	0	1	2a	2b
			proportion	0.96419	0.03581	0.00000	0.00000
			background ω	0.02195	1.00000	0.02195	1.00000
			foreground ω	0.02195	1.00000	1.00000	1.00000
Megabats	64	−5322.454355	site class	0	1	2a	2b
			proportion	0.96419	0.03581	0.00000	0.00000
			background ω	0.02195	1.00000	0.02195	1.00000
			foreground ω	0.02195	1.00000	1.00000	1.00000
Megabats ω_m_ = 1	63	−5322.454355	site class	0	1	2a	2b
			proportion	0.96419	0.03581	0.00000	0.00000
			background ω	0.02195	1.00000	0.02195	1.00000
			foreground ω	0.02195	1.00000	1.00000	1.00000

Since all bats are nocturnal, we also tested the selective pressure on the branch of their common ancestor (marked *b* in Figure 5). Both the two-ratio and branch-site model fail to detect any evidence for positive selection. Megabats have a greater reliance on vision compared to microbats, thus we tested for selective pressures on the common ancestral branch for megabats (marked *m* in Figure 5). Again, we failed to detect positive selection. Likewise, the use of site models, with or without outgroups, also failed to detect evidence positive selection (Table 1). Similarly, we failed to detect any positive selection in *M/LWS* (Table S1). Although we failed to detect positive selection in the *RH1* gene, this does not mean that the *RH1* gene was not under adaptive evolution in bats, it result may simply be due to the limited power of the statistical methods [25].

Although bats are a monophyletic group, during their long evolutionary history (>52 million years) [26] they have become a very diverse group, with different diets, echolocating ability, vision, roosting habitats, body size, olfaction, and so on, to fit their own unique environments. Although bats are very diverse, all of them are nocturnal, however, their eyes differ dramatically (Figure 5) reflecting differences on their reliance on vision. Old-World fruit bats generally have large eyes and navigate by sight. Microbats have developed echolocation, and mostly use acoustic orientation (echolocation) rather than vision. Unlike most of the microbats that live in a completely dark environment and have degraded eyes, the tomb bat has relatively normal eyes and lives in places that are not necessarily shielded from light, such as among rocks, or hanging from trees, walls, or eaves and emerging before nightfall to hunt. It appears that the tomb bat, like Old-World fruit bats, don't dislike light as much as other microbats, and thus these two groups may rely more on dim-light vision, and have had convergent evolution of their *RH1* genes. The reamining parallel changes observed within bats may imply that during the long nocturnal history of bats that the dim-light vision gene may have been prone to convergences, possibly due to ecological specialization (i.e., various photic environments). Alternatively, some of these parallel changes, and reversals may reflect constrains upon the sequences, where two alternative amino acids may be tolerated at these locations. These results emphasize that further attention is needed on the functional characterization of these sequences and the ecology and behavior of bats.

Many vertebrates use vision as their principal means to interpret the environment, and have evolved a diversity of visual systems reflecting their adaptive responses to various types of light environments [1], [27]. In this study, we found that the dim-light vision gene (*RH1*) had undergone strong purifying selection in both microbats and megabats, revealing an important role for dim-light vision in their nocturnal lifestyle, despite microbats developing acoustic orientation (echolocation) and being thought to rely mostly on sonar rather than vision. Since sonar only works best over short distances, vision appears to be primarily used for the detection of landmarks and to avoid objects when moving over long distances, for example during seasonal migration or commuting between feeding sites [18]. The variable features of echolocation, such as frequency, bandwidth, duration and pulse interval are all related to the ecological niche of the bats [28]. Thus similarly to echolocation, the multiple parallel amino acid replacements in *RH1* suggest the possibility of multiple convergences of ecological specialization (such as various photic environments) during adaptation to the nocturnal lifestyle. However, we note, that the effects of the parallel sites on properties such as wavelength shifts needs experimental conformation. Therefore, future studies which should include greater taxon sampling and functional experiments should yield more insight into our conclusions.

A recent study found that the phylogenetic topology of the *Prestin* gene unites echolocating bats, a topology that differs from the species phylogeny, thus it was concluded that *Prestin* was subjected to convergent evolution while playing a role in the evolution of echolocation in bats [29]. Here, we found that the phylogenetic topology of *RH1* genes differs from the species topology of bats, uniting Old-World fruit bats and Yangochiroptera. Further analysis, however, revealed that multiple episodes of convergent evolution in the *RH1* gene of bats occurred, rather than just the simple convergence of Old-World fruit bats and Yangochiroptera, thus this study also emphasizes that careful attention to the complete phylogeny must be considered before concluding convergent evolution simply from a putative gene tree.

## Materials and Methods

### Ethics Statement

All research involving animals in this study follow the guidelines of the byelaw of experiments on animals, and have been approved by the Ethics and Experimental Animal Committee of Kunming Institute of Zoology, Chinese Academy of Sciences.

### Source of Data and Primary Treatments

Rhodopsin (*RH1*), short wave opsin (*SWS1*), and long/middle wave opsin (*M/LWS*) gene sequences of the little brown bat (*Myotis lucifugus*), flying fox (*Pteropus vampyrus*), cow and dog were downloaded from the Ensembl database. The cDNA sequences of the opsin genes from these species were aligned using CLUSTALX 1.81 [30]. Gene-specific primers were designed based on conserved regions (Table S2). A total of 23 bat individuals were determined for this study and analyzed together with other available sequences obtained from GenBank and Ensembl (Table S3).

### RNA Isolation and Sequencing

The 23 bat individuals (list in Table S3) were humanely killed. The eyes were excised rapidly and frozen in liquid nitrogen. Total RNA was isolated from the eyes using the RNAiso™ Plus Kit (Takara, China), and stored at −80°C. RT-PCR was performed on 2 µg RNAs using the PrimeScript™ RT-PCR Kit (Takara, China) to attain cDNA and opsin genes were amplified from the cDNA using gene-special primers (Table S2). PCR amplifications were carried out using the following touchdown program: 95°C 4 min, 20 cycles of 94°C denaturation 1 min, 60–50°C annealing (1 min; −0.5°C/cycle), 72°C extension 1 min, and finally 15 cycles of 94°C 1 min, 50°C 1 min, 72°C 1 min. PCR products were cleaned using the Watson PCR Purification Kits (Watson BioTechnologies, Shanghai).

Each PCR product was sequenced at least three times on an ABI 3730 Sequencer (Applied Biosystems, Foster, CA, USA) using the ABI PRISM BigDye Terminator v3.0. DNA sequences were edited using DNAstar Seqman software (DNASTAR Inc., Madison, WI, USA) and the newly determined sequences were deposited in GenBank (Accession numbers GQ863406-GQ863461).

### Phylogenetic and Molecular Evolutionary Analysis

For each gene, nucleotide sequences were translated into amino acid sequences and aligned using CLUSTALX 1.81 [30]. Alignments were visually checked for accuracy and used as a guide for the alignment of the nucleotide sequences for evolutionary analyses. The best fit model of nucleotide evolution was determined by Modeltest [31], and Maximum Likelihood trees was reconstructed by PAUP [32] and Bayesian phylogenies was revealed by MrBayes [33].

We used the Li-Wu-Luo method [34] to reconstruct a NJ (Neighbor-Joining) tree based on synonsymous and nonsynonymous sites. In this method, each site in a codon is allocated to a 0-fold, 2-fold or 4-fold degenerate category. For computing distances, all 0-fold and two-thirds of the 2-fold sites are considered nonsynonymous, whereas one-third of the 2-fold and all of the 4-fold sites are considered synonymous.

Tests for selection and ancestor sequence reconstruction were carried out using the Codeml program implemented in PAML [35], [36]. The same suite of tests was conducted for the *RH1* and *M/LWS* genes: (1) one-ratio model, which assumes an identical ω value for all branches, where ω is the ratio of nonsynonymous to synonymous substitution rates; (2) a free-ratio model, assuming an independent ω values for each branch, to provide a rough measure of the selective pressure on each branch; (3) two-ratio model and (4) branch-site model were used to determine whether these genes have undergone positive selection on a foreground branch; (5) site models: the neutral model (M1a) estimates two ω values (0<ω_0_<1, ω_1_ = 1); the positive selection model (M2a) adds an extra ωvalue to M1a; M8 (β &ω model) takes into account the possibility of positively selected (PS) sites; and M8a is the null model of M8. Bayes Empirical Bayes (BEB) analysis was used to calculate the Bayesian posterior probability of PS sites. Finally, LRT statistics were calculated between following model pairs: (1) the two-ratio model vs. the one-ratio model were compared to test whether the ω ratio is significantly different from that of other mammals; (2) test 1 (branch-site model vs. site model M1a) and test 2 (branch-site model vs. branch-site model with fixed ω_1_ = 1) for branch-site model [37] were conducted; (3) M1a vs. M2a and M8 vs. M8a were compared to examine possible positive selection sites. In the previous cases, twice the difference in log-likelihood values (2ΔlnL) between the two models was calculated following a chi-squared (χ2) distribution with the degrees of freedom equaling the difference in the number of parameter estimated for the model pairs.

## Supporting Information

Figure S1 The aligned sequences of M/LWS gene in bats and their divergent sites.
(8.74 MB TIF)

Figure S2 Phylogenetic tree based on M/LWS opsin gene. Numbers above the branches are the ML bootstrap values, while numbers under the branches are the Bayesian posterior probabilities.
(0.23 MB TIF)

Figure S3 Phylogenetic tree based on SWS1 opsin gene. Numbers above the branches are the ML bootstrap values, while numbers under the branches are the Bayesian posterior probabilities.
(2.38 MB TIF)

Table S1 Selective pressure analyses on M/LWS gene.
(0.06 MB DOC)

Table S2 The primers for amplifying *RH1*, M/LWS and SWS1 opsin genes.
(0.04 MB DOC)

Table S3 Summary of sequences surveyed in this study.
(0.08 MB DOC)
