# Supplementary figures and images for: Parallel and Convergent Evolution of the Dim-Light Vision Gene RH1 in Bats (Order: Chiroptera)
Source: PLoS One. 2010 Jan 21;5(1):e8838. doi: 10.1371/journal.pone.0008838 (PMC2809114; doi:10.1371/journal.pone.0008838)

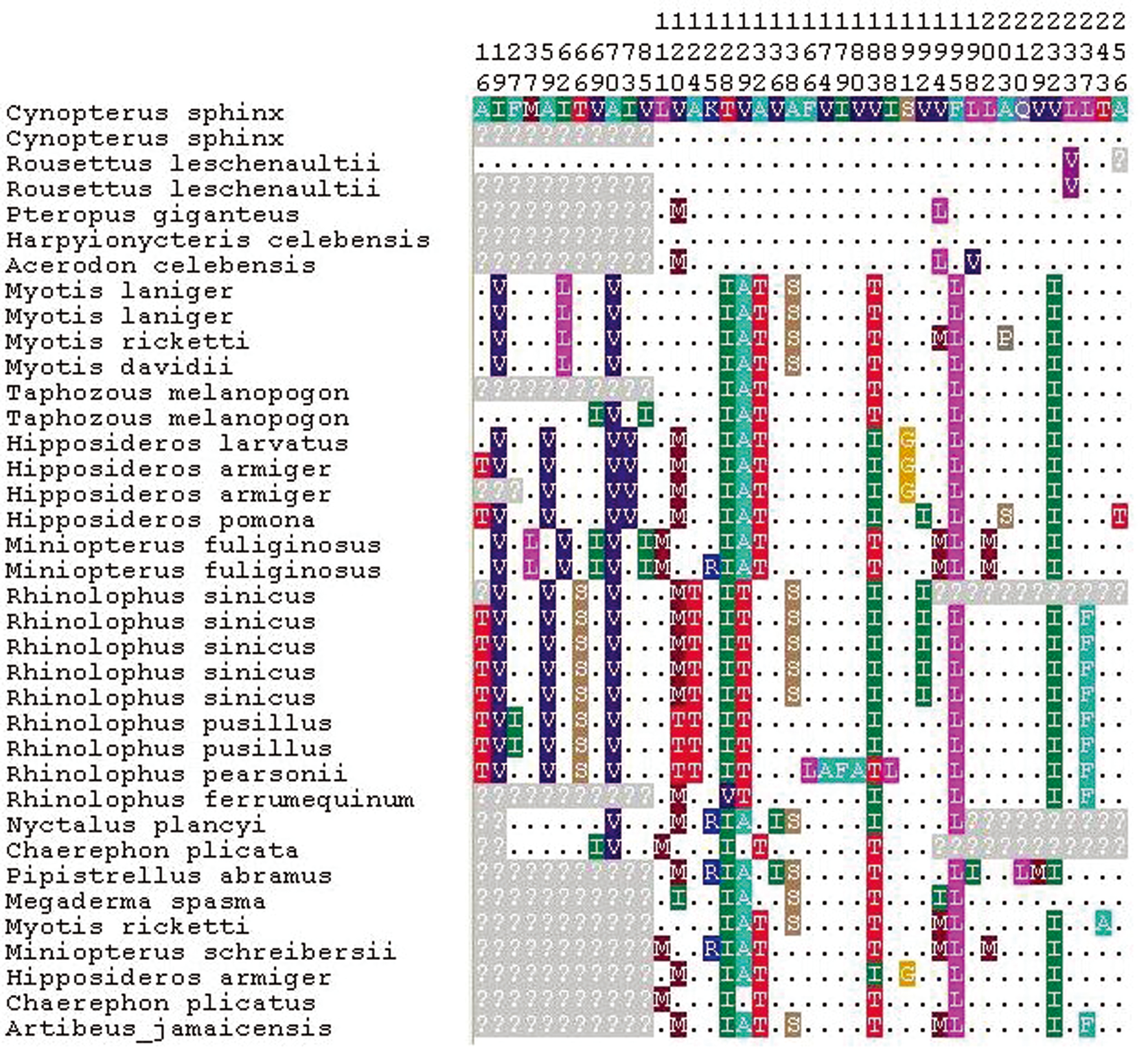

Supplement: Figure S1 — The aligned sequences of M/LWS gene in bats and their divergent sites. (8.74 MB TIF) [file pone.0008838.s001.tif]

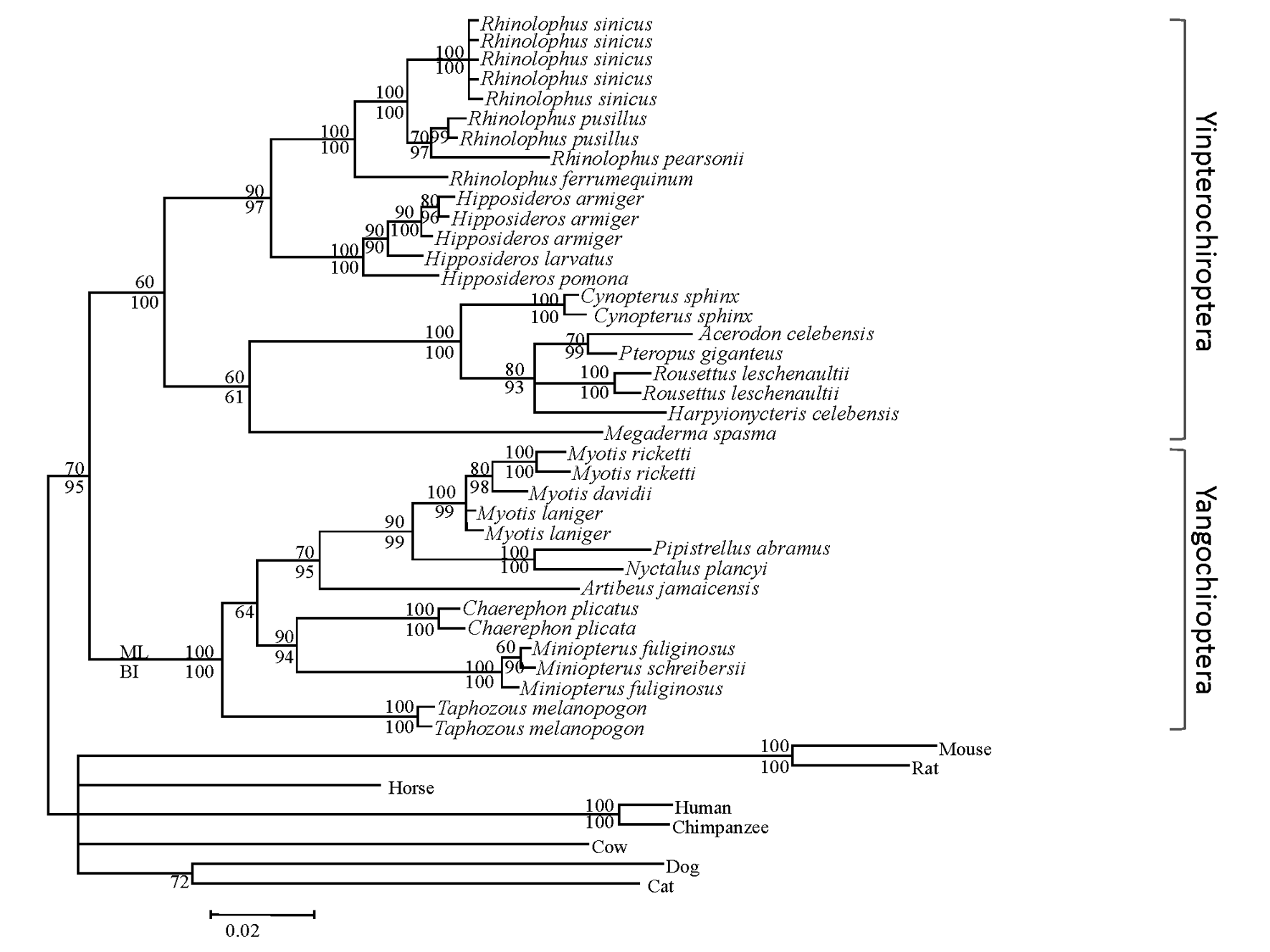

Supplement: Figure S2 — Phylogenetic tree based on M/LWS opsin gene. Numbers above the branches are the ML bootstrap values, while numbers under the branches are the Bayesian posterior probabilities. (0.23 MB TIF) [file pone.0008838.s002.tif]

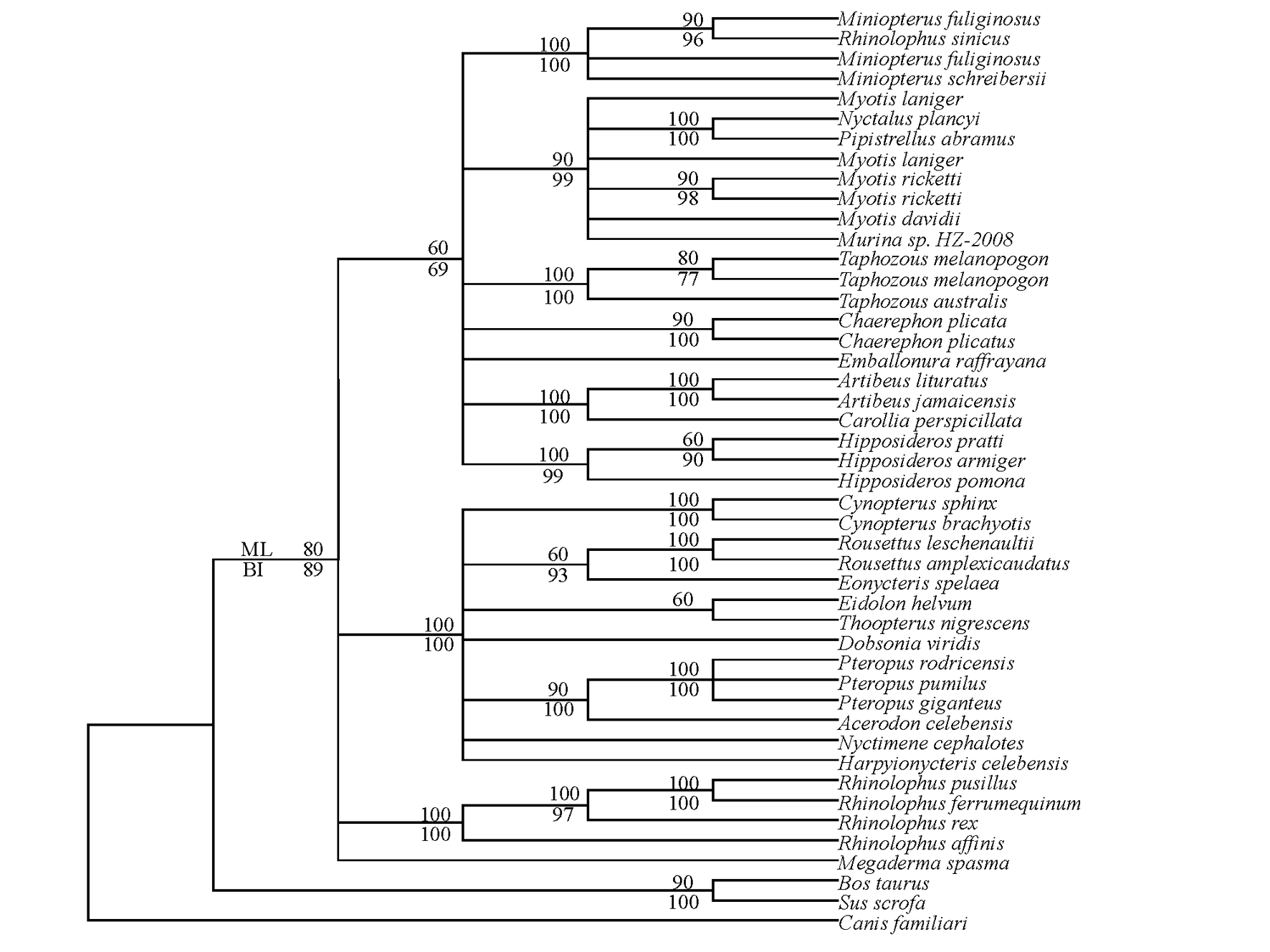

Supplement: Figure S3 — Phylogenetic tree based on SWS1 opsin gene. Numbers above the branches are the ML bootstrap values, while numbers under the branches are the Bayesian posterior probabilities. (2.38 MB TIF) [file pone.0008838.s003.tif]
