# Supplementary material for: Parallel and Convergent Evolution of the Dim-Light Vision Gene RH1 in Bats (Order: Chiroptera)
Source: PLoS One. 2010 Jan 21;5(1):e8838. doi: 10.1371/journal.pone.0008838 (PMC2809114; doi:10.1371/journal.pone.0008838)
Supplement: Table S1 — Selective pressure analyses on M/LWS gene. (0.06 MB DOC) [file pone.0008838.s004.doc]

Table S1:

Selective pressure analyses on *M/LWS* gene.

| Model | P | Ln L | Estimates of parameters |
| --- | --- | --- | --- |
| M0: one ratio | 82 | -4237.065053 | ω=0.0731 |
| Two ratio: |  |  |  |
| The common ancestor of bats | 83 | -4241.514793 | ω_b_=0.1704, ω_0_=0.0723 |
| The common ancestor of bats ω_b_=1 | 82 | -4242.827880 | ω_0_=0.0717 |
| Megabats | 83 | -4241.969906 | ω_b_=0.0728, ω_0_=0.0733 |
| Megabats ω_m_=1 | 82 | -4259.030520 | ω_0_=0.0723 |
| Site models (only contain bats) |  |  |  |
| M1a | 69 | -2800.509852 | p: 0.95005 0.04995 |
|  |  |  | ω: 0.04209 1.00000 |
| M2a | 71 | -2800.509852 | p: 0.95005 0.04734 0.00261 |
|  |  |  | ω: 0.04209 1.00000 1.00000 |
| M8 | 71 | -2796.668594 | p_0_=0.97228 p=0.24550 q=4.10660 |
|  |  |  | (p_1_=0.02772) ω=1.06440 |
| M8a | 70 | -2796.642332 | p_0_=0.96963 p=0.23807 q=4.00135 |
|  |  |  | (p_1_=0.03037) ω=1.00000 |
| Branch-site models |  |  |  |
| The common ancestor of bats | 85 | -4192.778983 | site class 0 1 2a 2b |
|  |  |  | proportion 0.84459 0.06236 0.08666 0.00640 |
|  |  |  | background ω 0.03825 1.00000 0.03825 1.00000 |
|  |  |  | foreground ω 0.03825 1.00000 1.00000 1.00000 |
| The common ancestor of bats ω_b_=1 | 84 | -4192.778983 | site class 0 1 2a 2b |
|  |  |  | proportion 0.84459 0.06236 0.08666 0.00640 |
|  |  |  | background ω 0.03825 1.00000 0.03825 1.00000 |
|  |  |  | foreground ω 0.03825 1.00000 1.00000 1.00000 |
| Megabats | 85 | -4193.184791 | site class 0 1 2a 2b |
|  |  |  | proportion 0.92848 0.07152 0.00000 0.00000 |
|  |  |  | background ω 0.03822 1.00000 0.03822 1.00000 |
|  |  |  | foreground ω 0.03822 1.00000 1.00000 1.00000 |
| Megabats ω_m_=1 | 84 | -4193.184791 | site class 0 1 2a 2b |
|  |  |  | proportion 0.92848 0.07152 0.00000 0.00000 |
|  |  |  | background ω 0.03822 1.00000 0.03822 1.00000 |
|  |  |  | foreground ω 0.03822 1.00000 1.00000 1.00000 |
