# Supplementary material for: Parallel and Convergent Evolution of the Dim-Light Vision Gene RH1 in Bats (Order: Chiroptera)
Source: PLoS One. 2010 Jan 21;5(1):e8838. doi: 10.1371/journal.pone.0008838 (PMC2809114; doi:10.1371/journal.pone.0008838)
Supplement: Table S2 — The primers for amplifying RH1, M/LWS and SWS1 opsin genes. (0.04 MB DOC) [file pone.0008838.s005.doc]

Table S2: The primers for amplifying *RH1*, *M/LWS* and *SWS1* opsin genes.

| Primer name | Primer sequence (5' to 3') | Ta (℃) |
| --- | --- | --- |
| *RH1* |  |  |
| RHO_S71 | TCGAGTAYCCGCAGTAC | Touchdown 60-50 |
| RHO_A1037 | TTCTTGCCACAGCAGAG |  |
| RHO_S46 | AAGACGGGCGTGGTGC | Touchdown 60-50 |
| RHO_A894 | GTGGGTRAAGATGTAGAATGC |  |
| *M/LWS* |  |  |
| RSO_S73 | GCGAGCATCTTCAYCTAC | 48 |
| RSO_ A1024 | CCACYTTCTTCCCAAAA |  |
| RSO_ S131 | ATTACCACATCGCYCCC | 55 |
| RSO_ A764 | TCTTTCTGCTGCTTYGC |  |
| *SWS1* |  |  |
| BSO_ S65 | GGGAYGGGCCTCAGTAC | Touchdown 60-50 |
| BSO_ A945 | GATGCARGCCCGGAAC |  |
| BSO_ S140 | TTGYAGGGACRCCACT | Touchdown 60-50 |
| BSO_ A938 | GCYCGGAACTGCTTATT |  |
