# Supplementary material for: Parallel and Convergent Evolution of the Dim-Light Vision Gene RH1 in Bats (Order: Chiroptera)
Source: PLoS One. 2010 Jan 21;5(1):e8838. doi: 10.1371/journal.pone.0008838 (PMC2809114; doi:10.1371/journal.pone.0008838)
Supplement: Table S3 — Summary of sequences surveyed in this study. (0.08 MB DOC) [file pone.0008838.s006.doc]

Table S3: Summary of sequences surveyed in this study.

| Taxonomy  (order) | Species name | *SWS1* opsin | *M/LWS* opsin | *Rhodopsin* |
| --- | --- | --- | --- | --- |
| Carnivora | *Canis lupus* | XM_539386 | XM_538203 | X71380 |
|  | *Felis catus* | ENSFCAG00000000952 | NM_001009871 | NM_001009242 |
| Cetartiodactyla | *Bos taurus* | NM_174567 | NM_174566 | NM_001014890 |
| Artiodactyla | *Sus scrofa* | AY091587 | NM_174566 | NM_214221 |
|  | *Equus caballus* | ENSECAG00000023205 | AF132043 | ENSECAG00000013709 |
| Chiroptera | *Myotis lucifugus* | FJ002252 | FJ002251 | FJ002250 |
|  | *Pteropus vampyrus* | ENSPVAG00000001081 | ENSPVAG00000000474 | ENSPVAG00000007112 |
|  | *Cynopterus sphinx** | GQ863415 | GQ863439 | GQ863420 |
|  | *Rousettus leschenaulti** |  | GQ863440 | GQ863419 |
|  | *Chaerephon plicata** | GQ863412 | GQ863460 | GQ863421 |
|  | *Taphozous melanopogon** | GQ863411 | GQ863443 | GQ863424 |
|  | *Hipposideros larvatus** |  | GQ863445 | GQ863427 |
|  | *Hipposideros armiger** |  | GQ863461 | GQ863417 |
|  | *Hipposideros armiger** |  | GQ863448 | GQ863425 |
|  | *Hipposideros pomona** |  | GQ863454 | GQ863426 |
|  | *Myotis ricketti** | GQ863414 | GQ863444 | GQ863423 |
|  | *Nyctalus velutinus** | GQ863408 | GQ863459 | GQ863422 |
|  | *Rhinolophus sinicus** |  | GQ863455 | GQ863438 |
|  | *Rhinolophus sinicus** |  | GQ863458 |  |
|  | *Rhinolophus sinicus** |  | GQ863451 | GQ863437 |
|  | *Rhinolophus sinicus** | GQ863413 | GQ863446 | GQ863434 |
|  | *Rhinolophus sinicus** |  | GQ863449 | GQ863433 |
|  | *Rhinolophus pearsonii** |  | GQ863457 | GQ863432 |
|  | *Rhinolophus pusillus** |  | GQ863456 | GQ863429 |
|  | *Rhinolophus pusillus** |  | GQ863452 | GQ863430 |
|  | *Miniopterus fuliginosus** | GQ863406 | GQ863447 | GQ863431 |
|  | *Miniopterus fuliginosus** | GQ863410 | GQ863450 | GQ863418 |
|  | *Myotis davidii** | GQ863416 | GQ863453 | GQ863436 |
|  | *Myotis laniger** | GQ863409 | GQ863441 | GQ863428 |
|  | *Myotis laniger** | GQ863407 | GQ863442 | GQ863435 |
|  | *Miniopterus_schreibersii* | EU912376 | EU912344 |  |
|  | *Hipposideros_armiger* | EU912368 | EU912343 |  |
|  | *Cynopterus_sphinx* |  | EU912342 |  |
|  | *Chaerephon_plicatus* | EU912359 | EU912341 |  |
|  | *Artibeus_jamaicensis* | EU912367 | EU912340 |  |
|  | *Harpyionycteris_celebensis* | EU912356 | EU912339 |  |
|  | *Acerodon_celebensis* | EU912354 | EU912338 |  |
|  | *Eonycteris_spelaea* | EU912375 |  |  |
|  | *Rhinolophus_pusillus* | *EU912382* |  |  |
|  | *Murina_sp._HZ-2008* | EU912379 |  |  |
|  | *Emballonura_raffrayana* | EU912374 |  |  |
|  | *Eidolon_helvum* | EU912373 |  |  |
|  | *Artibeus_lituratus* | EU912372 |  |  |
|  | *Dobsonia_viridis* | EU912371 |  |  |
|  | *Hipposideros_pomona* | EU912370 |  |  |
|  | *Hipposideros_pratti* | EU912369 |  |  |
|  | *Rhinolophus_rex* | EU912365 |  |  |
|  | *Pteropus_rodricensis* | EU912363 |  |  |
|  | *Pteropus_pumilus* | EU912362 |  |  |
|  | *Carollia_perspicillata* | EU912358 |  |  |
|  | *Nyctimene_cephalotes* | EU912357 |  |  |
|  | *Thoopterus_nigrescens* | EU912355 |  |  |
|  | *Cynopterus_brachyotis* | EU912353 |  |  |
|  | *Rousettus_amplexicaudatus* | EU912352 |  |  |

Note: *** species used in this study.
